# Influence of Keratoconus Severity on Detecting True Progression with Scheimpflug Imaging and Anterior Segment Optical Coherence Tomography

**DOI:** 10.3390/life13071474

**Published:** 2023-06-29

**Authors:** Sara Ortiz-Toquero, Carlota Fuente, Clara Auladell, Francisco Arnalich-Montiel

**Affiliations:** 1 Department of Ophthalmology, Ramón y Cajal University Hospital, Carretera de Colmenar Viejo Km 9, 100, 28034 Madrid, Spain; 2 Optometry Research Group, IOBA-Eye Institute, Department of Theoretical Physics, Atomic and Optics, University of Valladolid, 47011 Valladolid, Spain

**Keywords:** keratoconus, severity, Scheimpflug, anterior segment optical coherence tomography, progression, repeatability, reproducibility

## Abstract

To determine consistent change over time in keratoconus disease, it is necessary to establish progression cut-off values based on intersession variability of the device used to monitor the cornea. The aim of this study was to analyze the accuracy of corneal parameters using Scheimpflug tomography and anterior segment optical coherence tomography in healthy and keratoconic eyes of varying severity to determine the cut-off values that indicate real progression. Three repeated measurements of each cornea of healthy (20 eyes) and keratoconic eyes (mild = 16, moderate = 25 and severe = 20) were recorded using Pentacam and Casia SS-1000 devices, which were repeated 2–3 weeks later. K1, K2, maximal anterior and posterior keratometry, and corneal thickness at the thinnest location (TCT) were collected. The accuracy was excellent with both devices; however, the Casia device presented better repeatability and reproducibility in all parameters in all groups compared to the Pentacam. The cut-off of the Pentacam and Casia in the mild stage were lower (K1 = 0.50 and 0.37 D; K2 = 0.51 and 0.37 D; Kmax-A = 1.24 and 0.65 D; Kmax-P = 0.38 and 0.17 D; TCT = 19.64 and 11.19 µm) than that of the severe stage (K1 = 1.09 and 0.88 D; K2 = 1.41 and 0.87 D; Kmax-A = 2.74 and 2.15 D; Kmax-P = 0.82 and 0.22 D; TCT = 28.68 and 14.83 µm). These results show that the greater the keratoconus severity, the greater the change that must occur for it to be considered real.

## 1. Introduction

Keratoconus is a bilateral and asymmetric corneal disorder characterized by progressive thinning and steepening of the cornea [1,2]. This disease induces high myopia and irregular astigmatism, resulting in impairment of the quality of vision. Keratoconus affects between 50 and 230 subjects per 100,000 [1,2] and commonly appears during the second decade of life and puberty; it progresses until the fourth decade of life, when it usually stabilizes [1,2].

The recent development of new treatments and corneal imaging techniques has improved the management and follow-up of keratoconic eyes [3]. In 2003, corneal collagen cross-linking (CXL) was clinically introduced as a novel treatment to halt progression in patients who have progressive disease [4,5]. Early detection of progression in keratoconus patients is essential in monitoring and planning CXL treatment [1,5].

Emerging technologies, such as corneal Scheimpflug tomography or swept-source anterior segment optical coherence tomography (SS-OCT), have helped in the diagnosis of keratoconus and enhanced the ability to detect progression of the disease [3,6,7]. The *Global Consensus on Keratoconus and Ectatic Diseases* published in 2015 argued that there is no clear definition of keratoconus progression [1]. However, the experts of the global consensus determined that there is progression when at least two of the three following situations occur: consistent changes in the anterior and posterior curvature of the cornea and corneal thinning [1]. Furthermore, for the change to be considered consistent or real over time, it must be greater than the variability of the measurement of the device used to monitor the cornea [1]. Therefore, knowing the normal intersession reproducibility in keratoconic eyes is crucial to distinguishing random differences between measurements due to device noise from real corneal change and progression.

There are studies in the literature that show a better precision of corneal parameter measurements in healthy subjects than in patients with keratoconus [8,9,10,11]. Furthermore, it seems that in more advanced stages of keratoconus, these measurements have worse reliability, although the results are inconsistent and vary depending on the study and the technology analyzed [12]. To determine the real values of change in keratoconus disease, it is necessary to establish progression cut-off values based on intersession variability. However, as far as we know, there are no published studies proposing cut-off values based on intersession reproducibility indicative of progression according to the degree of severity of keratoconus using current corneal imaging techniques.

For this reason, the aim of this study was to analyze the intrasession repeatability and intersession reproducibility of the measurements of corneal parameters using Scheimpflug tomography and SS-OCT in a sample of keratoconic eyes of varying severity to determine the cut-off values that indicate a possible real progression of the disease.

## 2. Materials and Methods

### 2.1. Patients

This prospective, clinic-based observational study was performed at the Department of Ophthalmology, University Hospital Ramón y Cajal of Madrid (Spain) and included healthy subjects and keratoconus patients. All patients were examined by a corneal specialist (F.A.-M.) who confirmed the diagnosis of keratoconus after a complete eye examination, which included corneal tomography analysis and anterior eye biomicroscopy assessment. A randomly selected single eye was chosen from each subject for study in the control group, but there was no restriction on selecting both eyes in the keratoconus groups as it is a disease of asymmetric evolution [13]. The study was approved by the hospital’s ethics committee, and it adhered to the tenets of the Declaration of Helsinki. Written informed consent was obtained from each subject. Keratoconus eyes were divided into three stages of severity based on the Amsler–Krumeich classification [14]: mild (stage 1 of Amsler–Krumeich classification), moderate (stage 2) and severe (stages 3–4).

Patients with any active ocular surface disease, corneal opacities, glaucoma, use of medication that could affect ocular physiology, a history of any type of ocular surgery or corneal astigmatism greater than 2.00 diopters (D) (except in the keratoconus group) were excluded. Patients with severe scarring due to keratoconus were also excluded. The control patients showed a corrected distance visual acuity of 20/20 and refractive errors of ±3.00 diopters. Contact lens wear was discontinued for at least 2 weeks before the eye examination.

### 2.2. Devices

Scheimpflug images were taken using a Pentacam (software version 1.20r 112; Oculus Optikgeräte GmbH, Wetzlar, Germany). It is a noninvasive (contact-free) anterior segment topographer. The Scheimpflug camera and a short-wavelength slit light (blue-light-emitting diode with a wavelength of 475 nm) rotate together around the optical axis of the eye. The system rotates 180 degrees in approximately 2 s and captures 25 Scheimpflug slit images drawn in a concentric fashion and covering white-to-white.

SS-OCT images were acquired using a Casia SS-1000 (software version 6R.2, Tomey, Nagoya, Japan). This device is based on swept-light source Fourier-domain OCT technology. This system achieves high-resolution imaging (axial 10 mm; transversal 30 mm) with a swept-source laser wavelength of 1310 nm. It analyzes anterior and posterior corneal surfaces with white-to-white cross-sectional images captured in a concentric fashion (similar to the slit images with the Pentacam).

### 2.3. Measurement Procedure

Three separate measurements of each cornea were taken with the Scheimpflug camera and SS-OCT following the manufacturers’ guidelines in a darkened room after verification of instrument calibration. Measurements with both devices were performed on all patients in a randomized order and in a sequential order. The subject placed their chin on the chin rest and the forehead was pressed against the forehead strap. The measurement was performed after an eye blink when the eye was aligned to the visual axis. When necessary, the eyelids were gently opened by an assistant, always avoiding any pressure on the globe. The patients moved their chin from the chinrest between scans to eliminate the interdependence of successive measurements. Only scans with a quality specification of “OK” were used for analysis. The same procedure was repeated after 2 or 3 weeks by the same operator to determine the intersession reproducibility. All measurements were recorded between 9 AM and 1 PM to resemble the usual clinical conditions by a single examiner.

We collected various parameters from both devices and both sessions to determine the progression of ectatic disease including keratometric (K) readings in the flat (K1) and steep meridians (K2), maximal anterior (Kmax-A) and posterior keratometry (Kmax-P), and corneal thickness at the thinnest location (TCT).

### 2.4. Statistical Analysis

Statistical analyses were performed using SPSS for Windows software (version 24.0; SPSS, Inc., Chicago, IL, USA) and Microsoft Office Excel (Microsoft Corp. Washington, USA). To assess the normal distribution of the variables, the Kolmogorov–Smirnov test was used (*p* > 0.05 indicates that the data are normally distributed). Descriptive results are given as the mean and standard deviation (SD).

This study followed the definitions of intrasession repeatability and intersession reproducibility according to the British Standards Institute and the International Organization for Standardization [15]. To calculate the intersession repeatability, the following parameters were obtained from two repeated measurements of the first session: within-subject standard deviation (Sw) [16]; repeatability limit (r = 2.77 × Sw, which defines the difference between two measurements of the same patient for 95% of pairs of observation) [16]; coefficient of variation (CV; percentage value of the measurement’s variation and defined as the ratio of the Sw to the overall mean [CV = Sw/mean × 100 (%)]) [16]; and the intraclass correlation coefficient (ICC; classified as follows: less than 0.75 = poor agreement; 0.75 to less than 0.90 = moderate agreement; 0.90 or greater = high agreement) [17]. The limits of agreement (LoAs) were also calculated as the mean difference ±1.96 SD between measurements [18]. The benefit of CV values is that they can be compared between data sets with different units or extensively different means.

Intersession reproducibility was assessed using the first measurement of each session (Method 1: first measurement of Session 1 vs. first measurement of Session 2) and using the mean of three repeat measurements from each session (Method 2). The 95% LoAs were defined as the mean difference ±1.96 SD between measurements performed during the 2 different sessions [18]. The within-subject standard deviation (SR), reproducibility limit (R = 2.77 × SR), CV and ICC coefficients were also calculated. The cut-off value for the progression in keratoconic eyes was calculated by the upper limit of the 95% confidence interval of the 95% LoA.

Age was compared between groups using an ANOVA test (*p* < 0.05 considered statistically significant). Pearson’s correlation was used to determine the relationship between age and reproducibility coefficients (SR and CV; *p* < 0.05 considered statistically significant). The reproducibility coefficients between men and women were also compared using the Mann–Whitney U test (*p* < 0.05 considered statistically significant).

## 3. Results

### 3.1. Patient Demographics

Eighty-one eyes were included in the study. The control group comprised 20 eyes from 20 healthy patients (7 women, 13 men) with a mean age of 33.5 ± 9.6 years (range: 19 to 52 years). The keratoconus group comprised 61 eyes from 43 keratoconus patients (13 women, 30 men) with a mean age of 34.5 ± 13.0 years (range: 20 to 66 years). Considering the degree of severity, 16 eyes were included in the mild stage, 25 eyes in the moderate stage and 20 eyes in the severe stage. Supplementary Table S1 shows the gender and age distribution in each study group. No statistically significant differences were found in the mean age between control and keratoconus severity groups (*p* = 0.18).

### 3.2. Intrasession Repeatability

Table 1 shows the intrasession repeatability results for corneal parameters in healthy and keratoconic eyes. Repeated-measures ANOVA did not detect significant differences between the consecutive measurements for any parameter (*p* ≤ 0.05). Markedly, ICCs for all the parameters analyzed were above 0.98, which indicates excellent repeatability. Casia SS-1000 presented better intrasession repeatability values in all parameters in control eyes (CVs ≤ 0.18), and in mild (CVs ≤ 0.35), moderate (CVs ≤ 0.35) and severe (CVs ≤ 0.56%) stages of keratoconus compared to the Pentacam system (CVs ≤ 0.52%; ≤0.88%; ≤1.36% and ≤1.41%, respectively). The intrasession repeatability results were good with both devices, with the Kmax-P measurements with the Pentacam in the severe stage presenting the worst results (CV = 1.41%). There was a decrease in intrasession repeatability for both devices with a higher degree of severity of the disease.

### 3.3. Intersession Reproducibility

The intersession reproducibility was better for healthy than for keratoconic eyes with both devices (Table 2). The reproducibility results were also better with Casia than with Pentacam in all corneal parameters which decreased with increasing degree of severity of the disease.

When the mean of three repeated measurements (Method 2) instead of a single measurement (Method 1) was analyzed, the intersession reproducibility results improved in the control and keratoconic groups and for all parameters using the Casia (except KmaxP in control, mild and moderate eyes, as they were very similar) and Pentacam (Table 2).

This produced a mean reduction in the intersession R and CV values across all parameters in all groups with the Pentacam (R = 21.3%; range: 0 to 39%; CV = 20.5%; range: 7.8 to 40.3%) and Casia (R = 14.7%: range: 0 to 33.3%; CV = 13.8%; range: 0 to 45.2%) when the repeated measurements were used. Finally, the Kmax-P parameter presented the lowest reproducibility results with both the Pentacam (CVs ≥ 1.22) and Casia (CVs ≥ 0.39%).

No statistically significant correlation was found between the age of keratoconus patients and SR or CVs in either the total sample or in severity groups (Method 1 and 2; *p* ≥ 0.26). Moreover, no statistically significant differences were found between SR and CV in the total keratoconus sample between men and women (*p* = 0.13).

### 3.4. Cut-off Values to Consider a Corneal Shape Change

Table 3 reveals the cut-off values that indicate a possible progression of disease based on intersession reproducibility. In line with the results found for repeatability and reproducibility, the cut-off values were lower with the Casia than with the Pentacam system. Furthermore, it was found that, in severe keratoconus, the cut-off values for possible progression were higher than those in mild and moderate keratoconus. The cut-off values using the mean of three repeated measurements were globally reduced by 23.2% (range: 6.8% to 33.8%) and 13.3% (range: 0% to 37.4%) with the Pentacam and Casia, respectively, when compared to the cut-off values using a single measurement.

## 4. Discussion

Detecting progression is crucial in monitoring keratoconus to preserve the patient’s vision [4,5]. The *Global Consensus on Keratoconus and Ectasia Diseases* proposed CXL to treat progressive keratoconus to avoid or delay corneal keratoplasty [1]. However, they admitted that detecting progression can be challenging and difficult to define [1]. Various criteria can be applied as corneal indicators of progression, based on changes in anterior and/or posterior keratometry and decreases in corneal thickness [1,19,20]. Ferdi et al. [21] proposed that steeper K-Max, thicker TCT and younger age were the most clinically useful baseline predictors of progression and should be followed up more closely. On the other hand, Guber et al. [22] hypothesized that K1 and K2 are best positioned to detect changes in these patients. However, there are no consistent criteria for defining progression at the present time.

In our study, we have analyzed the intrasession repeatability and intersession reproducibility of two devices currently used in the monitoring of patients with keratoconus (Pentacam and Casia SS-1000) in a sample of healthy subjects and in patients with keratoconus with different degrees of severity. Threshold values for progression should exceed the normal noise of an imaging device [1]. As far as we know, this is the first study that proposes cut-off values as indicators of progression considering KC severity and intersession reproducibility.

Precision is defined as the result of variability detected with repeated measurements on the same subjects [13]. In this way, repeatability represents the minimum variability (factors held constant), and reproducibility represents the maximum variability (when one or more factors vary). In clinical practice, the eye care practitioner usually evaluates two examinations of a patient performed a few months apart to estimate whether there is progression of the keratoconus. Therefore, determining intersession reproducibility and calculating cut-off values based on the maximum possible variability is essential in the analysis of keratoconus progression.

Several studies have previously evaluated the accuracy of Scheimpflug and SS-OCT [6,9,11,12,22,23,24,25,26,27,28,29,30] in the setting of keratoconus, but without considering KC severity [6,9,11,24,27,28,30] or intersession reproducibility [6,9,11,12,22,23,24,25,26,27,28,29], which are important factors when studying topographic progression between two scans from different days. For example, De Luis Eguileor et al. [23] and Gustafsson et al. [29] have previously proposed cut-off values based on intersession repeatability (r), but this requires the minimum variability of the measurements to be analyzed. Moreover, Hashemi et al. [24] and Brunner et al. [6] proposed cut-off values based on interobserver repeatability, but they did not consider the degree of severity of the disease.

Consistent with other authors, we found the accuracy of both devices to be good in healthy subjects and patients with keratoconus [6,9,11]. However, we found that the reliability of both intrasession repeatability and intersession reproducibility worsened with increasing KC severity in line with previous articles using the Pentacam or Casia SS-1000 [12,23,25,26].

In our study, we observed better repeatability and reproducibility results using SS-OCT than with the Scheimpflug system. There are other studies that have compared SS-OCT and Scheimpflug with conflicting results. Brunner et al. [6] found better intersession and interobserver repeatability with the Pentacam HR compared to the Casia for K1, K2 and TCT in healthy and KC eyes. However, Szalai et al. [11] reported better repeatability results with the Casia in K1, K2 and apex corneal thickness compared to the Pentacam HR. However, we analyzed another version of Pentacam and the results are not comparable. Another study also found that Pentacam HR presented better reproducibility for the anterior corneal parameters, whereas Casia 2 showed better reproducibility for the posterior corneal parameters in healthy and KC eyes [25].

One of the most widespread criteria in the analysis of KC progression is the increase in the steepest K value (K1) by ≥1.0 D [31], which would be robust in indicating progression in mild and moderate cases but not in severe cases according to our results (intersession noise ≥1.25 D with the Pentacam and Casia with a single measurement). Epstein et al. [27] affirmed that when there are changes in the KMax-A between two scans separated in time that are greater than 1.51 D, this is considered a real change with 95% confidence in KC and post LASIK ectasias without distinction according to severity. For their part, Claesson et al. [30] assumed that an increase in Kmax-A of 1.0 D or more is defined as progression, regardless of the degree of severity. In our study, the intersession noise for KMax-A was greater than the threshold proposed by Claesson [30] (noise ≥1.04 D) and the progression criterion proposed by Epstein [27] in the moderate stage of KC with the Pentacam (1.74 D) and the severe stage with the Pentacam (3.18 D) and Casia (2.16 D). Therefore, it is crucial to carry out progression studies considering factors such as the severity of the disease. The progression criterion in mild stages of KC should be lower than that in advanced KC due to the variability in the measurements in these cases.

As evidenced in other studies [6,24,30] and in line with our results, the precision of the devices improves if the mean of the repeated measures is analyzed and, therefore, the cut-off values for progression decreases. For example, in our results, the cut-off value for K1 in moderate stages of KC increased if a single measurement was analyzed versus the mean value of three measurements using the Pentacam (0.78 versus 0.54 D; 30.8%) and Casia (0.70 versus 0.53 D; 24.3%). Brunner et al. [6] found a mean reduction in their cut-off values of progression of KC that was greater than ours when using the mean of three measurements for both the Pentacam (52.4% versus 21.3%) and Casia (46.4% versus 13.3%). In another study by Hashemi et al. [24], it was reported that when the mean of two measurements was used, the CR improved by 20% on average; however, it is not possible to improve this reduction by analyzing the mean of three measurements. In summary, the method used to examine progression in keratoconus by eye care practitioners, that is, single versus repeated measurements, may have a great influence on the decision to perform CXL treatment.

This study has some limitations. Only virgin keratoconus corneas have been included, but there is no evidence of progression values in patients undergoing CXL, intracorneal ring segments or keratoplasty. External factors could have significantly affected the accuracy of corneal measurements (e.g., saccadic eye movements, misalignment of patients’ heads in the forehead and chin rest, changes in tear film, etc.). However, a meticulously controlled data acquisition method could control the quality of the acquired images and minimize the effect of these external factors. The Pentacam device was used to calculate the new cut-off values, and it would be interesting to validate it against the current Pentacam HR version as there may be differences. Both eyes have been included in some of the keratoconus patients in the study and this may lead to a bias. However, as keratoconus is a bilateral but asymmetric disease, this was considered appropriate as in other published studies [9,11,25]. In addition, conducting a multicenter study to validate these cut-off values would be highly recommended, as well as the inclusion of other devices that are currently used to monitor the progression of keratoconus.

## 5. Conclusions

In this study, the cut-off values based on intersession reproducibility indicating possible progression with Scheimpflug and SS-OCT have been proposed for the first time in a sample including mild, moderate and severe stages of keratoconus. Our study reveals a real-life clinical impact on the management of keratoconus patients depending on which method of analysis (single vs. repeated measurements), corneal parameter and device is used as well as the degree of severity of the disease for detection of progression. Furthermore, these results show that the greater the corneal irregularity and thinning, the greater the change that must occur for it to be considered real and to overcome the variability of the device itself.

## Figures and Tables

**Table 1:** Intrasession repeatability in control and keratoconic (mild, moderate and severe stages) eyes for corneal parameters analyzed.

Parameter	Group	Mean ± SD	Sw	r	CV (%)	95% LOA	ICC	*p*-Value
PENTACAM								
K1 (D)	Control	43.29 ± 1.11	0.06	0.16	0.15	−0.29 to 0.27	0.99	0.76
	Mild	44.09 ± 1.90	0.10	0.28	0.23	−0.40 to 0.37	0.99	0.82
	Moderate	44.60 ± 2.98	0.13	0.36	0.30	−0.48 to 0.42	0.99	0.49
	Severe	47.27 ± 4.20	0.24	0.66	0.52	−0.86 to 0.85	0.99	0.96
K2 (D)	Control	44.09 ± 0.94	0.08	0.23	0.18	−0.32 to 0.36	0.99	0.61
	Mild	46.47 ± 2.03	0.12	0.33	0.26	−0.48 to 0.46	0.99	0.92
	Moderate	48.48 ± 2.47	0.14	0.39	0.29	−0.53 to 0.50	0.99	0.73
	Severe	51.41 ± 4.70	0.23	0.63	0.44	−1.07 to 0.75	0.99	0.14
Kmax-A (D)	Control	44.47 ± 1.15	0.10	0.28	0.18	−0.43 to 0.41	0.99	0.83
	Mild	49.03 ± 2.15	0.23	0.63	0.46	−0.95 to 0.79	0.98	0.48
	Moderate	53.64 ± 2.72	0.25	0.70	0.47	−1.09 to 0.80	0.98	0.18
	Severe	59.71 ± 5.73	0.29	0.81	0.49	−1.11 to 1.02	0.98	0.72
Kmax-P (D)	Control	−7.02 ± 0.83	0.03	0.08	0.39	−0.11 to 0.08	0.99	0.66
	Mild	−7.59 ± 0.54	0.07	0.20	0.88	−0.25 to 0.16	0.98	0.37
	Moderate	−8.57 ± 0.65	0.11	0.30	1.36	−0.50 to 0.35	0.96	0.12
	Severe	−9.76 ± 0.95	0.15	0.41	1.41	−0.54 to 0.58	0.96	0.64
TCT (µm)	Control	560.42 ± 31.78	2.90	8.02	0.52	−9.58 to 9.58	0.99	0.86
	Mild	495.00 ± 35.80	2.92	8.09	0.60	−10.82 to 9.82	0.99	0.71
	Moderate	480.33 ± 44.97	4.48	12.40	0.96	−15.87 to 15.70	0.99	0.96
	Severe	456.17 ± 34.47	5.11	14.15	1.13	−15.56 to 18.67	0.98	0.50
**CASIA SS-1000**								
K1 (D)	Control	43.16 ± 0.83	0.04	0.11	0.08	−0.16 to 0.11	0.99	0.98
	Mild	44.05 ± 1.90	0.06	0.16	0.14	−0.24 to 0.12	0.99	0.65
	Moderate	44.52 ± 2.70	0.12	0.33	0.27	−0.44 to 0.42	0.99	0.71
	Severe	46.24 ± 3.07	0.16	0.44	0.34	−0.61 to 0.72	0.99	0.32
K2 (D)	Control	43.71 ± 1.03	0.05	0.14	0.10	−0.20 to 0.11	0.99	0.75
	Mild	45.54 ± 2.04	0.05	0.15	0.11	−0.18 to 0.14	0.99	0.63
	Moderate	47.07 ± 2.21	0.12	0.33	0.25	−0.36 to 0.49	0.99	0.16
	Severe	49.46 ± 3.30	0.13	0.36	0.26	−0.36 to 0.50	0.99	0.11
Kmax-A (D)	Control	44.31 ± 0.89	0.08	0.22	0.18	−0.33 to 0.23	0.99	0.19
	Mild	47.83 ± 1.96	0.17	0.47	0.35	−0.65 to 0.56	0.99	0.44
	Moderate	51.62 ± 2.37	0.18	0.50	0.35	−0.47 to 0.76	0.99	0.14
	Severe	56.15 ± 4.39	0.23	0.63	0.39	−0.86 to 0.81	0.99	0.84
Kmax-P (D)	Control	−6.39 ± 0.16	0.01	0.02	0.13	−0.07 to 0.07	0.99	0.71
	Mild	−7.41 ± 0.56	0.02	0.05	0.24	−0.11 to 0.11	0.99	0.54
	Moderate	−8.14 ± 0.54	0.03	0.08	0.34	−0.15 to 0.11	0.99	0.14
	Severe	−9.06 ± 0.85	0.05	0.13	0.56	−0.24 to 0.23	0.99	0.85
TCT (µm)	Control	538.24 ± 28.66	0.75	2.07	0.13	−3.21 to 2.97	0.99	0.31
	Mild	482.50 ± 32.64	0.81	2.24	0.17	−3.32 to 3.63	0.99	0.19
	Moderate	466.88 ± 40.63	1.06	2.93	0.23	−3.79 to 4.79	0.99	0.27
	Severe	438.55 ± 32.62	1.08	2.99	0.26	−5.40 to 5.08	0.99	0.63

K1 = flat keratometry; K2 = steep keratometry; KMax-A = maximal anterior keratometry; KMax-P = maximal posterior keratometry; TCT = corneal thickness at the thinnest location; SD = standard deviation; Sw = within-subject standard deviation; r = limit of repeatability; CV = coefficient of variation; LoA = limits of agreement; ICC = intraclass correlation coefficient.

**Table 2:** Intersession reproducibility in control and keratoconic (mild, moderate and severe stages) eyes for corneal parameters analyzed (Method 1 and Method 2).

		Reproducibility (Method 1)							Reproducibility (Method 2)						
Parameter	Group	SD of Diff	SR	R	CV (%)	95% LOA	ICC	*p*-Value	SD of Diff	SR	R	CV (%)	95% LOA	ICC	*p*-Value
**PENTACAM**															
K1 (D)	Control	0.16	0.10	0.27	0.23	−0.24 to 0.40	0.99	0.45	0.16	0.08	0.22	0.19	−0.26 to 0.35	0.99	0.90
	Mild	0.26	0.16	0.44	0.36	−0.55 to 0.48	0.99	0.58	0.19	0.10	0.27	0.23	−0.41 to 0.33	0.99	0.75
	Moderate	0.32	0.19	0.52	0.42	−0.7 to 0.55	0.99	0.27	0.24	0.16	0.44	0.36	−0.59 to 0.36	0.99	0.81
	Severe	0.51	0.28	0.77	0.58	−1.04 to 0.98	0.99	0.79	0.39	0.25	0.69	0.52	−0.78 to 0.76	0.99	0.84
K2 (D)	Control	0.23	0.13	0.36	0.30	−0.39 to 0.53	0.98	0.21	0.14	0.09	0.24	0.20	−0.23 to 0.33	0.99	0.19
	Mild	0.25	0.15	0.41	0.33	−0.46 to 0.54	0.98	0.71	0.19	0.12	0.33	0.26	−0.34 to 0.35	0.99	0.24
	Moderate	0.34	0.19	0.52	0.39	−0.77 to 0.55	0.98	0.12	0.23	0.13	0.36	0.30	−0.52 to 0.37	0.99	0.31
	Severe	0.67	0.29	0.80	0.57	−1.55 to 1.06	0.98	0.11	0.54	0.24	0.66	0.46	−1.15 to 0.97	0.99	0.46
Kmax-A (D)	Control	0.41	0.24	0.66	0.50	−0.83 to 0.78	0.98	0.82	0.27	0.16	0.44	0.36	−0.53 to 0.53	0.99	0.82
	Mild	0.47	0.26	0.72	0.52	−0.82 to 1.04	0.98	0.38	0.43	0.24	0.66	0.48	−0.84 to 0.84	0.99	0.74
	Moderate	0.67	0.38	1.05	0.71	−1.37 to 1.26	0.98	0.70	0.55	0.30	0.83	0.57	−1.09 to 1.07	0.99	0.55
	Severe	1.22	0.59	1.63	0.95	−2.58 to 2.19	0.98	0.48	1.00	0.49	1.35	0.81	−2.00 to 1.93	0.99	0.87
Kmax-P (D)	Control	0.06	0.03	0.08	0.51	−0.13 to 0.12	0.99	0.68	0.05	0.03	0.08	0.41	−0.09 to 0.11	0.99	0.33
	Mild	0.18	0.10	0.27	1.34	−0.44 to 0.27	0.97	0.15	0.15	0.09	0.24	1.22	−0.36 to 0.24	0.97	0.43
	Moderate	0.26	0.14	0.38	1.65	−0.50 to 0.53	0.96	0.66	0.17	0.09	0.24	1.45	−0.35 to 0.33	0.97	0.49
	Severe	0.30	0.17	0.47	1.71	−0.49 to 0.65	0.96	0.17	0.28	0.14	0.38	1.53	−0.51 to 0.59	0.97	0.54
TCT (µm)	Control	6.57	3.83	10.60	0.70	−12.72 to 13.03	0.98	0.60	4.54	2.62	7.25	0.47	−9.40 to 8.38	0.99	0.59
	Mild	6.85	3.67	10.16	0.74	−11.86 to 14.99	0.98	0.38	6.20	3.46	9.46	0.68	−10.42 to 13.88	0.98	0.34
	Moderate	12.96	7.13	19.75	1.50	−26.83 to 23.99	0.97	0.59	9.63	5.37	14.87	1.02	−20.60 to 17.13	0.98	0.39
	Severe	15.42	9.15	25.34	2.01	−28.06 to 32.39	0.95	0.52	10.21	5.58	15.45	1.20	−18.09 to 21.94	0.98	0.44
**CASIA SS-1000**															
K1 (D)	Control	0.10	0.05	0.13	0.12	−0.23 to 0.18	0.99	0.53	0.10	0.05	0.13	0.12	−0.20 to 0.18	0.99	0.34
	Mild	0.16	0.10	0.27	0.23	−0.40 to 0.23	0.99	0.08	0.15	0.08	0.22	0.18	−0.32 to 0.25	0.99	0.54
	Moderate	0.28	0.15	0.41	0.35	−0.60 to 0.50	0.99	0.27	0.21	0.12	0.33	0.26	−0.42 to 0.40	0.99	0.71
	Severe	0.43	0.21	0.58	0.47	−0.81 to 0.90	0.99	0.16	0.29	0.15	0.42	0.49	−0.49 to 0.65	0.99	0.07
K2 (D)	Control	0.14	0.09	0.24	0.20	−0.32 to 0.24	0.99	0.08	0.11	0.06	0.16	0.14	−0.24 to 0.20	0.99	0.10
	Mild	0.15	0.09	0.25	0.21	−0.36 to 0.24	0.99	0.34	0.15	0.10	0.27	0.20	−0.35 to 0.24	0.99	0.37
	Moderate	0.22	0.12	0.33	0.25	−0.51 to 0.36	0.99	0.10	0.20	0.10	0.28	0.21	−0.47 to 0.31	0.99	0.42
	Severe	0.39	0.24	0.66	0.49	−0.68 to 0.86	0.99	0.15	0.30	0.16	0.44	0.32	−0.57 to 0.62	0.99	0.09
Kmax-A (D)	Control	0.30	0.15	0.42	0.34	−0.64 to 0.55	0.98	0.51	0.20	0.11	0.30	0.22	−0.41 to 0.37	0.99	0.64
	Mild	0.35	0.20	0.55	0.42	−0.64 to 0.72	0.98	0.55	0.22	0.13	0.36	0.23	−0.42 to 0.43	0.99	0.62
	Moderate	0.47	0.24	0.66	0.46	−0.97 to 0.88	0.98	0.10	0.42	0.23	0.63	0.45	−0.90 to 0.74	0.99	0.22
	Severe	0.72	0.37	1.02	0.69	−1.26 to 1.57	0.98	0.21	0.71	0.36	0.99	0.68	−1.20 to 1.57	0.99	0.08
Kmax-P (D)	Control	0.06	0.02	0.05	0.29	−0.13 to 0.11	0.99	0.21	0.06	0.02	0.05	0.29	−0.14 to 0.10	0.99	0.13
	Mild	0.08	0.02	0.05	0.32	−0.17 to 0.14	0.98	0.17	0.06	0.02	0.05	0.32	−0.13 to 0.11	0.99	0.19
	Moderate	0.07	0.03	0.08	0.39	−0.15 to 0.12	0.98	0.20	0.07	0.03	0.08	0.38	−0.14 to 0.12	0.99	0.39
	Severe	0.15	0.08	0.22	0.68	−0.34 to 0.23	0.98	0.11	0.09	0.05	0.13	0.53	−0.23 to 0.14	0.99	0.42
TCT (µm)	Control	2.98	1.46	4.04	0.27	−6.03 to 5.67	0.99	0.75	2.44	1.36	3.76	0.25	−4.85 to 4.70	0.99	0.73
	Mild	3.71	2.37	6.56	0.50	−6.81 to 7.73	0.99	0.42	2.72	1.90	5.26	0.39	−4.41 to 6.25	0.99	0.41
	Moderate	4.67	2.74	7.58	0.60	−7.36 to 10.85	0.99	0.44	4.24	2.51	6.95	0.55	−6.61 to 10.00	0.99	0.25
	Severe	5.91	2.75	7.61	0.64	−12.42 to 10.94	0.99	0.45	5.42	2.74	7.58	0.63	−10.81 to 10.42	0.99	0.43

K1 = flat keratometry; K2 = steep keratometry; KMax-A = maximal anterior keratometry; KMax-P = maximal posterior keratometry; TCT = corneal thickness at the thinnest location; SD = standard deviation; SR = within-subject standard deviation; R = limit of reproducibility; CV = coefficient of variation; LoA = limits of agreement; ICC = intraclass correlation coefficient. Reproducibility Method 1 was calculated using the first measurement of each session and Method 2 with the mean of the three measurements made in each session.

**Table 3:** Summary of cut-off values for corneal consistent change in control and keratoconic eyes expressed as upper 95% LOAs with upper 95% CI of intersession reproducibility using the first measurement of each session (Method 1) and the mean of the three measurements of each session (Method 2).

Cut-Off for Corneal Consistent Change					
		PENTACAM		CASIA SS-1000	
Parameter	Group	Single Measurement (Method 1)	Repeated Measurements (Method 2)	Single Measurement (Method 1)	Repeated Measurements (Method 2)
K1 (D)	Control	0.53	0.48	0.26	0.25
	Mild	0.72	0.50	0.38	0.37
	Moderate	0.78	0.54	0.70	0.53
	Severe	1.40	1.09	1.25	0.88
K2 (D)	Control	0.72	0.44	0.35	0.29
	Mild	0.77	0.51	0.38	0.37
	Moderate	0.80	0.53	0.51	0.46
	Severe	1.60	1.41	1.17	0.87
Kmax-A (D)	Control	1.12	0.75	0.80	0.54
	Mild	1.48	1.24	1.04	0.65
	Moderate	1.74	1.46	1.22	1.04
	Severe	3.18	2.74	2.16	2.15
Kmax-P (D)	Control	0.17	0.15	0.15	0.15
	Mild	0.44	0.38	0.22	0.17
	Moderate	0.72	0.45	0.17	0.17
	Severe	0.88	0.82	0.35	0.22
TCT (µm)	Control	18.38	12.07	7.65	7.34
	Mild	21.36	19.64	11.19	8.78
	Moderate	33.26	24.01	14.29	13.03
	Severe	42.65	28.68	15.55	14.83

K1 = flat keratometry; K2 = steep keratometry; KMax-A = maximal anterior keratometry; KMax-P = maximal posterior keratometry; TCT = corneal thickness at the thinnest location.

## Data Availability

The data presented in this study are available on reasonable request from the corresponding author.

## Supplementary Materials

The following supporting information can be downloaded at: https://www.mdpi.com/article/10.3390/life13071474/s1, Table S1: Summary of the number of eyes/patients included in each group.

## References

[1] Gomes J.A., Tan D., Rapuano C.J., Belin M.W., Ambrósio R., Guell J.L., Malecaze F., Nishida K., Sangwan V.S., Group of Panelists for the Global Delphi Panel of Keratoconus and Ectatic Diseases (2015). Global consensus on keratoconus and ectatic diseases. Cornea.

[2] Romero-Jiménez M., Santodomingo-Rubido J., Wolffsohn J.S. (2010). Keratoconus: A review. Cont. Lens. Anterior Eye.

[3] Martínez-Abad A., Piñero D.P. (2017). New perspectives on the detection and progression of keratoconus. J. Cataract Refract. Surg..

[4] Mastropasqua L. (2015). Collagen cross-linking: When and how? A review of the state of the art of the technique and new perspectives. Eye Vis..

[5] Sykakis E., Karim R., Evans J.R., Bunce C., Amissah-Arthur K.N., Patwary S., McDonnell P.J., Hamada S. (2015). Corneal collagen cross-linking for treating keratoconus. Cochrane Database Syst. Rev..

[6] Brunner M., Czanner G., Vinciguerra R., Romano V., Ahmad S., Batterbury M., Britten C., Willoughby C.E., Kaye S.B. (2018). Improving precision for detecting change in the shape of the cornea in patients with keratoconus. Sci. Rep..

[7] Holladay J.T. (2009). Keratoconus detection using corneal topography. J. Refract. Surg..

[8] Ortiz-Toquero S., Rodriguez G., de Juan V., Martin R. (2014). Repeatability of placido-based corneal topography in keratoconus. Optom. Vis. Sci..

[9] de Luis Eguileor B., Escudero Argaluza J., Pijoán Zubizarreta J.I., Santamaria Carro A., Etxebarria Ecenarro J. (2018). Evaluation of the Reliability and repeatability of scheimpflug system measurement in keratoconus. Cornea.

[10] McMahon T.T., Anderson R.J., Roberts C., Mahmoud A.M., Szczotka-Flynn L.B., Raasch T.W., Friedman N.E., Davis L.J. (2005). Repeatability of corneal topography measurement in keratoconus with the TMS-1. Optom. Vis. Sci..

[11] Szalai E., Berta A., Hassan Z., Módis L. (2012). Reliability and repeatability of swept-source fourier-domain optical coherence tomography and scheimpflug imaging in keratoconus. J. Cataract Refract. Surg..

[12] Kreps E.O., Jimenez-Garcia M., Issarti I., Claerhout I., Koppen C., Rozema J.J. (2020). Repeatability of the Pentacam HR in various grades of keratoconus. Am. J. Ophthalmol..

[13] McAlinden C., Khadka J., Pesudovs K. (2015). Precision (repeatability and reproducibility) studies and sample-size calculation. J. Cataract Refract. Surg..

[14] Krumeich J.H., Daniel J., Knülle A. (1998). Live-epikeratophakia for keratoconus. J. Cataract Refract. Surg..

[15] McAlinden C., Khadka J., Pesudovs K. (2011). Statistical methods for conducting agreement (comparison of clinical tests) and precision (repeatability or reproducibility) studies in optometry and ophthalmology. Ophthalmic Physiol. Opt..

[16] Bland J.M. (2000). An Introduction to Medical Statistics.

[17] McGraw K.O., Wong S.P. (1996). Forming inferences about some intraclass correlation coefficients. Psychol. Methods.

[18] Bland J.M., Altman D.G. (1986). Statistical methods for assessing agreement between two methods of clinical measurement. Lancet.

[19] Ferdi A.C., Nguyen V., Gore D.M., Allan B.D., Rozema J.J., Watson S.L. (2019). Keratoconus natural progression: A systematic review and meta-analysis of 11 529 eyes. Ophthalmology.

[20] Wagner H., Barr J.T., Zadnik K. (2007). Collaborative Longitudinal Evaluation of Keratoconus (CLEK) study: Methods and findings to date. Cont. Lens Anterior Eye.

[21] Ferdi A., Nguyen V., Kandel H., Tan J.C.K., Arnalich-Montiel F.A., Abbondanza M., Watson S. (2022). Predictors of progression in untreated keratoconus: A Save Sight Keratoconus Registry Study. Br. J. Ophthalmol..

[22] Guber I., McAlinden C., Majo F., Bergin C. (2017). Identifying more reliable parameters for the detection of change during the follow-up of mild to moderate keratoconus patients. Eye. Vis..

[23] de Luis Eguileor B., Arriola-Villalobos P., Pijoan Zubizarreta J.I., Feijoo Lera R., Santamaria Carro A., Diaz-Valle D., Etxebarria J. (2021). Multicentre Study: Reliability and repeatability of Scheimpflug system measurement in keratoconus. Br. J. Ophthalmol..

[24] Hashemi K., Guber I., Bergin C., Majo F. (2015). Reduced precision of the Pentacam HR in eyes with mild to moderate keratoconus. Ophthalmology.

[25] Flockerzi E., Elzer B., Daas L., Xanthopoulou K., Eppig T., Langenbucher A., Seitz B. (2021). The reliability of successive scheimpflug imaging and anterior segment optical coherence tomography measurements decreases with increasing keratoconus severity. Cornea.

[26] Flynn T.H., Sharma D.P., Bunce C., Wilkins M.R. (2016). Differential precision of corneal Pentacam HR measurements in early and advanced keratoconus. Br. J. Ophthalmol..

[27] Epstein R.L., Chiu Y.L., Epstein G.L. (2012). Pentacam HR criteria for curvature change in keratoconus and postoperative LASIK Ectasia. J. Refract. Surg..

[28] Neuhann S., Schuh A., Krause D., Liegl R., Schmelter V., Kreutzer T., Mayer W.J., Kohnen T., Priglinger S. (2020). Comparison of variables measured with a Scheimpflug device for evaluation of progression and detection of keratoconus. Sci. Rep..

[29] Gustafsson I., Bergström A., Myers A.C., Ivarsen A., Hjortdal J. (2020). Association between keratoconus disease severity and repeatability in measurements of parameters for the assessment of progressive disease. PLoS ONE.

[30] Claesson M., Zetterberg M. (2018). Repeated same-day versus single tomography measurements of keratoconic eyes for analysis of disease progression. Cornea.

[31] Shetty R., Pahuja N.K., Nuijts R.M.M.A., Ajani A., Jayadev C., Sharma C., Nagaraja H. (2015). Current protocols of corneal collagen cross-linking: Visual, refractive, and tomographic outcomes. Am. J. Ophthalmol..

