# Supplementary material for: Influence of Keratoconus Severity on Detecting True Progression with Scheimpflug Imaging and Anterior Segment Optical Coherence Tomography
Source: Life (Basel). 2023 Jun 29;13(7):1474. doi: 10.3390/life13071474 (PMC10382025; doi:10.3390/life13071474)
Supplement: Supplementary file 1 [file life-13-01474-s001.zip › life-2427793-supplementary.pdf]

Supplementary Table S1

Group	N eyes / N patients	Mean age ± SD (range)	Gender (Men/Women)
Control	20 / 20	33.5 ± 9.6 (19 to 52)	13 / 7
All keratoconus	61 / 43	34.5 ± 13.0 (20 to 66)	30 / 13
Mild KC	16 / 16	34.9 ± 12.5 (20 to 61)	13 / 3
Moderate KC	25 / 22	33.4 ± 9.9 (20 to 60)	16 / 6
Severe KC	20 / 16	33.9 ± 11.7 (20 to 66)	11 / 5
